# Incidence and Pattern of Hypertensive Disorders of Pregnancy in Akwa Ibom and Cross River States, Nigeria

**DOI:** 10.7759/cureus.100467

**Published:** 2025-12-31

**Authors:** Saturday Etuk, Chinyere J Akpanika, Anietimfon S Akpan, Mbereobong S Etuk, Komommo Okpebri, Solomon Akpaka, Victor B Eyo, Ubong B Akpan, Soter Ameh, Oliver C Ezechi

**Affiliations:** 1 Department of Obstetrics and Gynecology, University of Calabar, University of Calabar Teaching Hospital, Calabar, NGA; 2 Department of Obstetrics and Gynecology, University of Calabar Teaching Hospital, Calabar, NGA; 3 Department of Obstetrics and Gynecology, University of Uyo Teaching Hospital, Uyo, NGA; 4 Department of Community Medicine, University of Calabar, University of Calabar Teaching Hospital, Calabar, NGA; 5 Department of Clinical Sciences, Nigerian Institute of Medical Research, Lagos, NGA

**Keywords:** akwa ibom state, cross river state, hypertensive disorders of pregnancy, incidence, pattern, prevalence

## Abstract

Introduction: Hypertensive disorders of pregnancy (HDP) are the leading cause of maternal and perinatal morbidity and mortality in developing countries. The incidence and pattern of these disorders are not known in most of these countries. Hence, the need to determine the incidence and pattern of these disorders in Akwa Ibom State (AKS) and Cross River State (CRS) of Nigeria.

Methods: In a prospective cohort study, all pregnant women who presented at the booking clinics of the referral level hospitals in AKS and CRS over a ten-month period (February 2023 to November 2023) were screened. Those with a confirmed gestational age of ≤20 weeks, whether hypertensive or normotensive, who gave their informed consent were recruited for the study and followed up till delivery. Data collected included age, parity, gestational age at enrollment, educational status, and marital status. Booking Blood pressure, weight, and height were measured, and qualitative urine protein status was also obtained for each participant. The data obtained were analyzed using STATA (version 16, StataCorp, TX, USA).

Results: Of the 1,006 participants that were followed up till delivery in this study, 25 had chronic hypertension, 240 had pre-eclampsia, and 24 had gestational hypertension. Those married were 967/1,006 (96.1%) and 447/1,006 (44.4%) were in private businesses. The majority of them had up to secondary 322/1,006 (32%) or tertiary 652/1,006 (64.8%) level of education. The period prevalence of HDP in the study population was 28.7% (289/1,006) and 2.5% (25/1,006) for chronic hypertension. The incidence of HDP was 26.9% (264/981); 24.5% (240/981) for pre-eclampsia and 2.4% (24/981) for gestational hypertension in the study population. The patterns of HDP were 2.5% (25/1,006) for chronic hypertension; 23.9% (240/1,006) for pre-eclampsia; and 2.4% 24/1,006) for gestational hypertension in the study population.

Conclusion: The incidence and prevalence of HDP in both AKS and CRS are high. Pre-eclampsia is the most common form of HDP in these states. There is a need for thorough screening for HDP in our antenatal clinics, identification of risk factors, and implementation of strategies to improve the outcomes of HDP in these states.

## Introduction

Hypertensive disorders of pregnancy (HDP) are a multisystem disease. They affect about 5-10% of pregnancies around the world and are an important cause of maternal and perinatal morbidity and mortality [1]. About 30,000 maternal and 500,000 perinatal deaths that occur every year can be ascribed to HDP [2]. It is the most common indication for admission of obstetric patients to the intensive care unit (ICU) [3]. The adverse outcomes of these disorders are disproportionally distributed across the world, with sub-Saharan Africa bearing the greatest burden [4]. The World Health Organization (WHO) estimates that the incidence of pre-eclampsia, a component of hypertensive disorders of pregnancy, is about seven times higher in developing countries than in the developed world [5]. The risk of a woman in a low-income country dying from pre-eclampsia/eclampsia is 300 times that of a woman in a high-income country. In some African settings, HDP has overtaken hemorrhage as the leading cause of maternal death.

According to the American College of Obstetricians and Gynecologists, the classification of hypertensive disorders of pregnancy is dependent on the gestational age at which the diagnosis of hypertension is first made, and twenty weeks of gestation remains the cut-off point used [6]. This classification is essential as the different types of HDP have different levels of risk and degrees of complications for both mother and baby. Classification, also facilitates communication among practitioners [7], enabling researchers to compare results, share a common language, and guide authors and editors on the presentation of results and outcomes. The various types of hypertensive disorders of pregnancy include: chronic hypertension or pre-existing hypertension, which occurs if the hypertension pre-dates pregnancy or occurs before 20 completed weeks of pregnancy in the absence of neoplastic trophoblastic disease [6]. In this type of HDP, the hypertension also continues after the pregnancy. Gestational hypertension, another form of HDP, refers to hypertension that occurs after the 20th week of pregnancy in the absence of proteinuria or any end-organ dysfunction, in a previously normotensive woman [8]. The maternal and perinatal risks associated with gestational hypertension are dependent on the gestational age at which it develops and the progression to pre-eclampsia. It has been noted that when gestational hypertension appears before 34 weeks of pregnancy, up to 35% of the patients develop pre-eclampsia, which raises the risk of complications to both the mother and her fetus [9,10], and that it takes an average of five weeks for pre-eclampsia to develop in this group of patients [11]. Pre-eclampsia, another type of HDP, is a pregnancy-specific disorder defined as gestational hypertension with proteinuria or typical end-organ dysfunction [7]. It is generally classified into mild and severe. Severe pre-eclampsia can be defined by blood pressure values greater than or equal to 160 mmHg systolic and/or 110 mmHg diastolic. Here, the amount of proteinuria is not considered mandatory [12]. Multi-organ involvement can also be used as the basis for the definition of severe pre-eclampsia, or the association with one or more severe complications, including stillbirth. Eclampsia, one of the most serious complications of pre-eclampsia, is defined as the occurrence of one or more generalized tonic-clonic convulsions and/or coma that are unrelated to other medical conditions during pregnancy or following delivery in women with hypertensive disorders of pregnancy [13,14]. Chronic hypertension with superimposed pre-eclampsia is another form of HDP. It is said to occur in a woman whose hypertension predates the current pregnancy or who was diagnosed before 20 completed weeks of pregnancy who then develops worsening high blood pressure and proteinuria or other health complications [15].

In 2022, Tukur et al. [16], in analyzing the quality and outcomes of maternal and perinatal care for 76,563 pregnancies from prospective data on women and their babies during pregnancy and delivery in 54 referral level hospitals in Nigeria, showed that eclampsia, a complication of HDP, was the most common cause of maternal and perinatal death. This work also specifically revealed HDP as the most common cause of maternal death in the South-South geo-political zone of Nigeria, where Akwa Ibom and Cross River States are situated, contributing 19.6%, about nine times the contribution of postpartum hemorrhage (2.2%) to these deaths [16]. Despite these negative impacts of HDP on the lives of pregnant women and their fetuses, there has been no current study on the subject. The few available ones are old and are single-center studies. Besides, the incidence and pattern of HDP are not yet established in Akwa Ibom and Cross River States despite these findings. Hence, the aim of this study was to establish the prevalence, incidence, and pattern of HDP in Akwa Ibom and Cross River States, as this may help to unfold the magnitude of the problem and possibly the need to work towards the prevention of these disorders and mitigation of their impacts on our mothers and their babies.

## Materials and methods

This study was conducted in Akwa Ibom and Cross River States, which are in the South-South geo-political zone of Nigeria.

Akwa Ibom State

Akwa Ibom State has a projected population of 6.50 million as of 2023. It has three referral level hospitals taking care of pregnant women: the University of Uyo Teaching Hospital (UUTH), Saint Luke Hospital, Anua (SLHA), and Police Hospital, Ikot Akpan Abia (PHI).

UUTH is a tertiary hospital and offers clinical training for University of Uyo medical students and is also an accredited center for postgraduate medical training. Its department of obstetrics and gynecology has 120 beds and offers antenatal care services to an average of 1,600 pregnant women and 1,100 deliveries a year. The hospital receives referrals from general hospitals, private hospitals, and primary health care facilities. Referrals also come from neighboring States of Cross River, Abia, and Rivers states.

SLHA is a secondary faith-based health facility. It offers antenatal care services to about 2,300 pregnant women and 1,300 deliveries a year and receives referrals from public and private health facilities in the neighborhood. It has 101 beds for obstetric and gynecological care.

PHI is a secondary health facility and has a busy obstetrics and gynecology department that offers antenatal care services to about 3,700 pregnant women and 1,400 deliveries in a year. It receives referrals from public and private health facilities in the neighborhood.

Cross River State

The projected population of Cross River State as at 2023 was about 4.4 million. It also has three referral level hospitals, the University of Calabar Teaching Hospital (UCTH), the Nigerian Navy Reference Hospital (NNRH), and the General Hospital, Calabar (GHC).

UCTH is the only tertiary health facility in Cross River State. It is the clinical training facility for the University of Calabar medical students. It is also an accredited center for postgraduate medical training. The department of obstetrics and gynecology has 93 beds and offers antenatal care services to about 1,700 pregnant women and delivery services to 1,050 pregnant women a year. UCTH receives referrals from all the secondary health facilities, primary health centers, and private clinics in the state. It also receives referrals from the neighboring states of Akwa Ibom and Benue, as well as its neighboring country, the Republic of Cameroon.

GHC is a secondary health facility with a busy obstetrics and gynecology department that offers antenatal care services to about 1,400 pregnant women and 1,250 deliveries in a year. The obstetric unit has 21 beds. It receives referrals from private clinics and primary health centers in the city.

NNRH is a secondary health facility with a department of obstetrics and gynecology that offers antenatal care services for about 930 pregnant women and 790 deliveries a year. It has 22 beds for obstetric patients. It receives referrals from primary health facilities and private clinics in the city.

The six referral level hospitals, three in each state, were used for this study. These referral level hospitals still practice the traditional model of antenatal care, where pregnant women with no complications are given appointments every four weeks from booking till 28 weeks, then every two weeks till 36 weeks, and subsequently every week till delivery.

Study design and sample size estimation

A prospective cohort study design was used to conduct this study. The sample size for the study was calculated using an online sample size calculator for cohort studies [17], and using prevalence rates of hypertension in pregnant women with and without risk for hypertension in south-west Nigeria (7.2%), and a dropout rate of 20%. The calculated sample size was 1,062 pregnant women with and without risk exposure to hypertension.

Study population

All pregnant women presenting at the booking clinics of the six referral level hospitals were screened for the study. All those who presented at ≤20 weeks’ gestation, were sure of their last menstrual period or had evidence of ultrasound-confirmed gestational age in the first trimester, were willing to deliver in any of the six hospitals or accept home visits and/or receive phone calls and gave their informed consent were recruited for the study.

The number of participants recruited from each center, as guided by the number of pregnant women offered antenatal care services a year in the center, was as follows: in Akwa Ibom State, 113, 160, and 260 were recruited from UUTH, SLHA, and PHI, respectively, giving a total number of 533. In Cross River State, 224, 185, and 122 were recruited from UCTH, GHC, and NNRH, respectively, giving a total number of 531. Therefore, the total number of participants from both states was 1,064.

Awareness and training of doctors, nurses and midwives in the antenatal clinics, antenatal wards, labor wards, and postnatal wards of the six hospitals involved in the caring for these obstetric patients were conducted. Two willing doctors from each of the study sites were selected and given a two-day physical training and monthly virtual training on the administration of the case record form designed for the study, as well as measurement of blood pressure, weight, height, and urine testing in this study.

This study lasted over a ten-month period (February 2023 to November 2023). The data collected included age, parity, date of enrollment, gestational age at enrollment, educational status of the woman and her partner, history of smoking, alcohol, history of previous marriage, and history of previous miscarriages. Age at marriage, history of infertility, previous history of hypertensive disorders of pregnancy and family history of multiple pregnancy, and diabetes mellitus were also obtained. Examination was done to establish the booking blood pressure, weight, and height, and qualitative urine protein status was obtained for each participant. 

With a standard scale (Seca GmbH & Co. KG, Hamburg, Germany), participants were weighed fully clothed with shoes removed, and height was also measured without shoes using a stadiometer (Seca GmbH & Co. KG, Hamburg, Germany) to the nearest centimeter. Qualitative urine protein was assessed using a dip stick (Uripath 2 brand, by Antec diagnostics Limited, Irvine, CA, USA) on a clean catch urine sample in a universal bottle by inserting a dip stick into the urine, and the result was read after about 60 seconds by comparing it with the standard on the dip stick container. The readings were as follows: negative, trace, + (30 mg/dl), ++ (100 mg/dl), +++ (300 mg/dl), and ++++ (2000 mg/dl or more). Qualitative urine protein reading of ++ or more was defined as significant proteinuria.

The participants were allowed to rest for about three to five minutes before the blood pressure was measured while seated on a chair. For the purpose of this study, hypertension was defined as blood pressure of ≥140/90 mmHg. The blood pressure measurement was done after ensuring that no tight clothing constricted the arm and that the non-dominant arm was well supported at the level of the heart on a table. The mercury sphygmomanometer cuff (Accoson brand) was placed on the arm with the center of the bladder over the brachial artery, and the lower edge of the cuff was placed 2-3 cm above the point of the pulsation of the brachial artery. The sphygmomanometer bladder cuff was made to encircle at least 80% of the participant’s arm. The radial artery was palpated to estimate the systolic blood pressure at the point where the pulsation disappeared while inflating the cuff. The cuff was then deflated, and the systolic blood pressure was again estimated at the point where the pulsation started. The sphygmomanometer was then re-inflated to about 30 mmHg above the estimated systolic blood pressure. A stethoscope diaphragm was then placed over the brachial artery while deflating the cuff at a rate of 2-3 mmHg/second until a regular tapping sound was heard. The systolic blood pressure was read at the point when the first sound was heard (Korotkoff phase I), while the diastolic blood pressure was taken at the point when the sound disappeared (Korotkoff phase V), and this was measured to the nearest 2 mmHg.

The participants’ follow-up was arranged to fit into the standard routine antenatal clinic visits. At each follow-up visit, the blood pressure, weight, and urine protein estimations were assessed and recorded for each of the participants. As these continued, those who developed HDP were noted and were still followed up till delivery. All the information was recorded in the case record form (CRF).

Data management and analysis

Data collected were entered into Microsoft Excel (Microsoft Corporation, Redmond, Washington, USA), and later exported to STATA (version 16.0, StataCorp, College Station, TX, USA) for analysis after data cleaning. The sample estimates of the prevalence and incidence of HDP were reported in percentages. To describe the presentation of HDP, summary frequency tables of sociodemographic characteristics, presentation time, clinical history, and clinical features were generated for the study population and by states. In addition, a summary tally of patients who presented by weeks across one full pregnancy term was generated to describe the HDP presentation pattern. Data were described in the general population and for both AKS and CRS.

Ethical considerations

Approval for the study was obtained from the Hospital Ethics Committees of the University of Calabar Teaching Hospital, Calabar, University of Uyo Teaching Hospital, Uyo, and also from the State Ministry of Health Ethics Review Committee of Akwa Ibom State and that of Cross River State, Approval Number UCTH/HREC/33/Vol.III/037. Written informed consent was obtained from each participant. For participants with a low level of literacy, assistance was sought from an available individual who was not a member of the research team.

## Results

**Figure 1:**
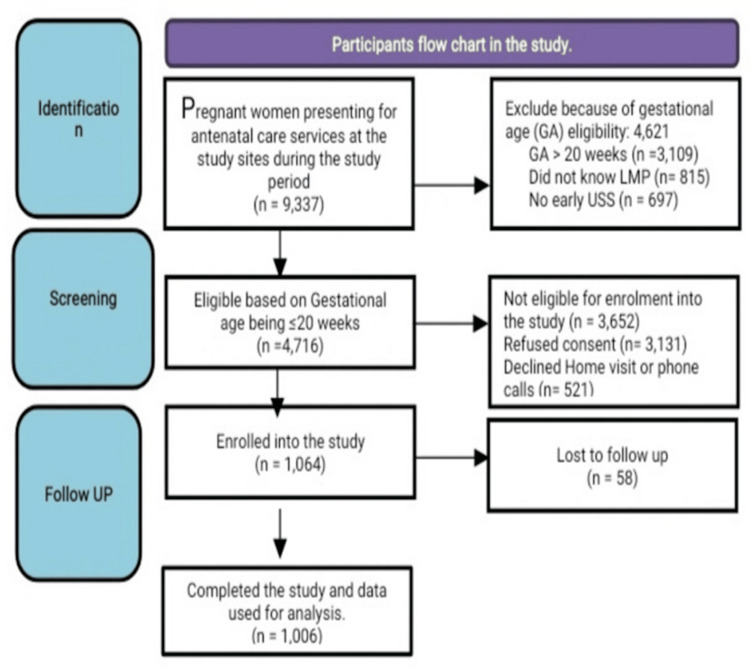
The STROBE flow diagram. STROBE: Strengthening the reporting of observational studies in epidemiology.

A total of 9,337 pregnant women who registered for antenatal care at the study sites during the study period were screened for inclusion into the study. Of these, 1,064 (11.4%) of them, whose gestational age was ≤20 weeks, were sure of their last menstrual period and/or had their first trimester ultrasound scan dating of the pregnancy, and also gave their informed consent, were enrolled in the study. At the end of the follow-up period, 1,006 (94.5%) out of the enrolled 1064 pregnant women completed the study and were included in the final analysis (see Figure 1).

Table 1 shows the sociodemographic characteristics of the study participants. The age of the participants in the study ranged from 16 to 50 years, with a mean age of 29.7 ± 4.9 years. Participants in the age group 20-30 years constituted the majority 589/1,006 (58.5%) of the study population. Most of them 967/1,006 (96.1%) were married or cohabiting. For over two-thirds 693/1006 (68.8%), the duration of cohabitation was for less than five years. Almost all the participants 990/1006 (98.4%) were Christians. A total of 392/1,006 (39.0%) of the participants were of the Ibibio tribe and 123/1006 (12.2%) were the Efiks. In terms of occupation, 447/1006 (44.4%) were doing their private businesses, 177/1,006 (17.6%) were civil servants, and 105/1,006 (10.4%) were traders. The majority of the participants had up to secondary 322/1,006 (32.0%) or tertiary 652/1,006 (64.8%) level of education. Most of the partners of the participants 721/1,006 (71.7%) had up to a tertiary level of education and had a mean age of 37.29 ± 5.50 years.

**Table 1:** Sociodemographic characteristics of the study participants.

Variables	Frequency (N = 1006)	Percentages
Age groups		
<20	14	1.4
20-30	589	58.5
>30	403	40.1
Marital status		
Single	39	3.9
Married/cohabiting	967	96.1
Religion		
Christianity	990	98.4
Islam	15	1.5
Others	1	0.1
Tribe		
Efik	123	12.2
Ibibio	392	39.0
Annang	96	9.5
Yakurr	86	8.5
Others	309	30.7
Occupation		
Business	447	44.4
Civil servant	177	17.6
Trading (small scale)	105	10.4
Teaching	68	6.8
Students	94	9.3
Applicants	102	10.1
Others	13	1.3
Level of education		
None	1	0.1
Primary	31	3.1
Secondary	322	32.0
Tertiary	652	64.8

Table 2 shows the obstetric and socio-behavioral characteristics of the participants in the study. The parity of the participants ranged from 0 to 10, with a mean of 1.48 ± 1.44. While the majority 580/1,006 (57.6%) of the participants had one 275/1,006 (27.3%) or no 305/1,006 (30.3%) previous delivery, the others either had 2-4, 380/1,006 (37.8%) or five and above 46/1,006 (4.6%) previous deliveries. Only 118/1,006 (11.7%) of the participants indulged in the use of alcohol, and 8/1,006 (0.8%) of them smoked cigarettes. Positive family history of multiple pregnancy 231/1,006 (23.0%) and previous miscarriages 363/1,006 (36.1%) were also reported by participants in the study.

**Table 2:** Obstetric and social behavioral characteristics of the participants in the study.

Variables	Frequency (N = 1006)	Percentages
Parity		
0	305	30.31
1	275	27.3
2-4	380	37.8
≥5	46	4.6
Smoking		
Yes	8	0.8
No	998	99.2
Alcohol intake		
Yes	118	11.7
No	888	88.3
Family history of multiple pregnancy		
Yes	231	23.0
No	775	77.0
History of previous miscarriages		
Yes	363	36.1
No	643	63.9

**Figure 2:**
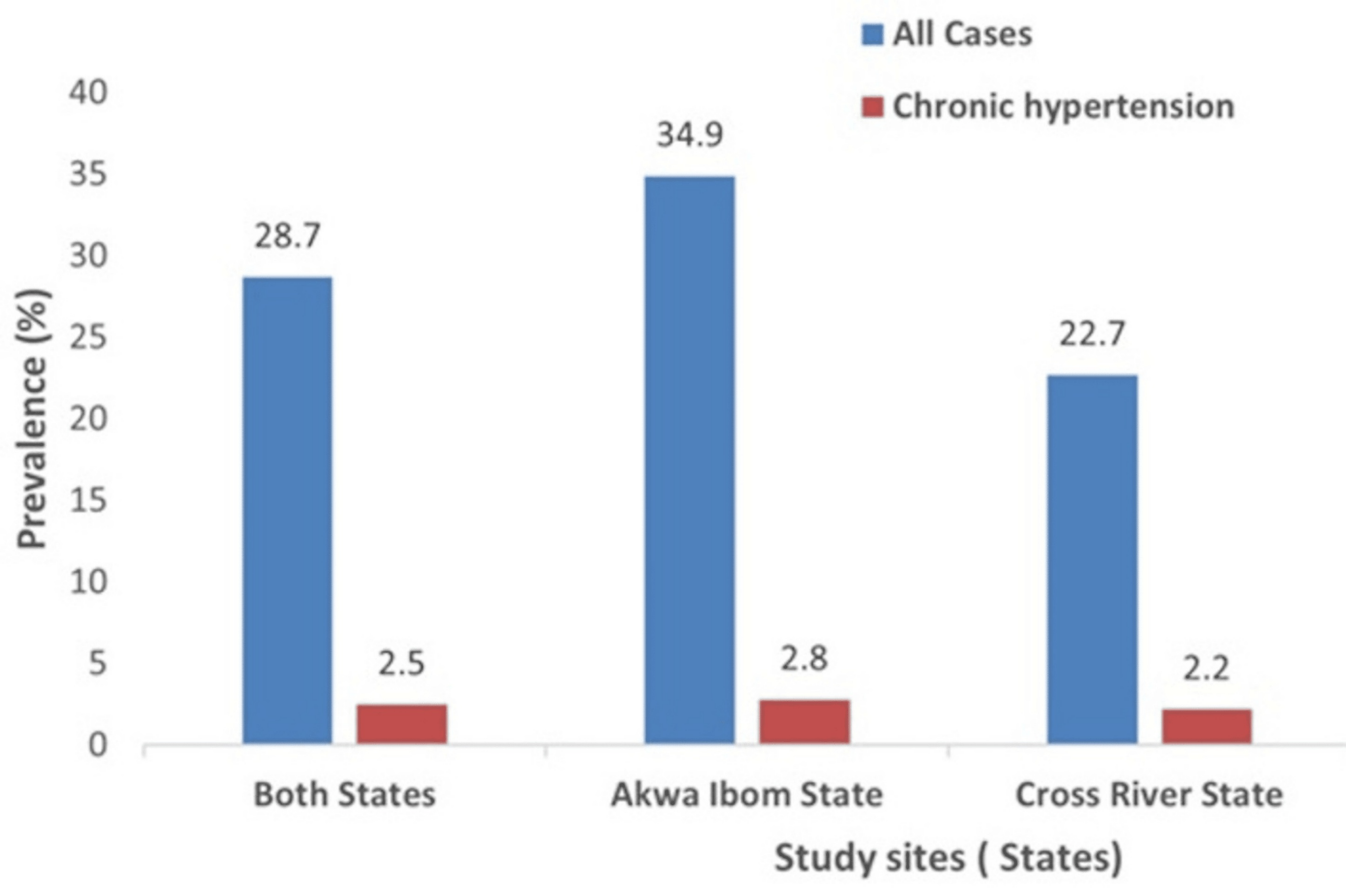
Prevalence of hypertensive disorders of pregnancy among participants in the study.

Figure 2 shows the prevalence of hypertension among the participants in the study. Of the 1,006 participants who completed the study follow-up, 496 (49.3%) of them were from Akwa Ibom State and 510 (50.7%) from Cross River State. Hypertension was diagnosed in 289 of the participants; 173 from Akwa Ibom State and 116 from Cross River State, giving a prevalence of hypertension of 28.7% (289/1,006) in the study population, 34.9% (173/496) in Akwa Ibom State, and 22.7% (116/510) in Cross River State. Twenty-five (25) of the hypertensive participants (14 from Akwa Ibom State and 11 from Cross River State) were previously diagnosed before the current pregnancy, giving a prevalence of chronic hypertension of 2.5% (25/1,006) in the study population, 2.8% (14/496) in Akwa Ibom State, and 2.2% (11/510) in Cross River State.

**Figure 3:**
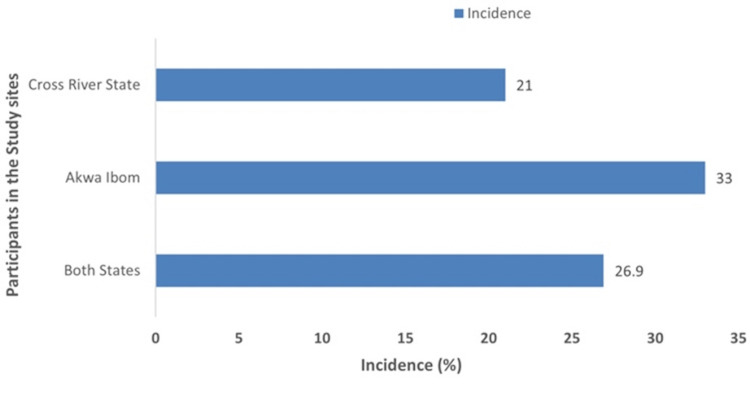
Incidence of hypertensive disorders in pregnancy among participants in the study.

The incidence of hypertension among the study participants and by state is shown in Figure 3. Among the 1,006 participants enrolled and followed up till discharge from the clinic in both AKS and CRS, 264 of them were diagnosed with hypertension for the first time in the current pregnancy or puerperium, giving an incidence of 26.9% (264/98). Of the 496 participants followed up till discharge in AKS, 159 were diagnosed with hypertension for the first time in the current pregnancy or puerperium; an incidence of 33.0% (159/482). Among the 510 participants enrolled and followed up till discharge from the clinics in CRS, 105 were diagnosed with hypertension for the first time in the current pregnancy or puerperium; an incidence of 21.0% (105/499).

**Figure 4:**
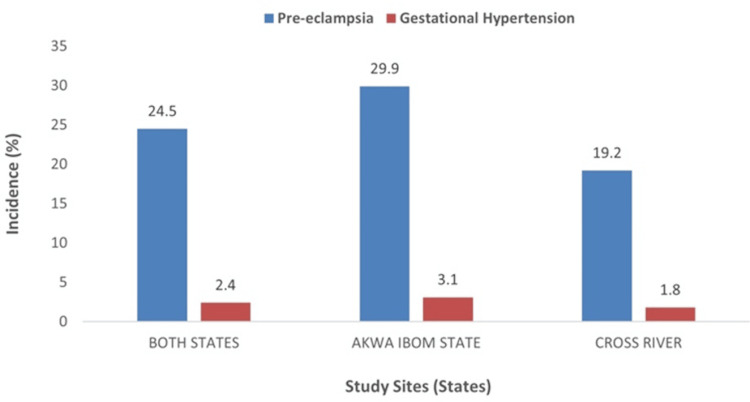
Incidence of pre-eclampsia and gestational hypertension in the study.

The incidence of pre-eclampsia and gestational hypertension among the participants in the study population and by states is shown in Figure 4. Of the 264 participants first diagnosed with hypertension in both states, 159 were from AKS and 105 from CRS; and 240; 144 and 96 of them from the study population, AKS and CRS respectively, were newly diagnosed with pre-eclampsia giving an incidence of 24.5% (240/981) in both states, 29.9% (144/482) in AKS and 19.2% (96/499) in CRS. Gestational hypertension was diagnosed in 24 participants in the study; 15 and nine participants respectively in both states, AKS and CRS; an incidence of 2.4% (24/981) in both states, 3.1% (15/482) in Akwa Ibom State, and 1.8% (9/499) in Cross River State.

**Figure 5:**
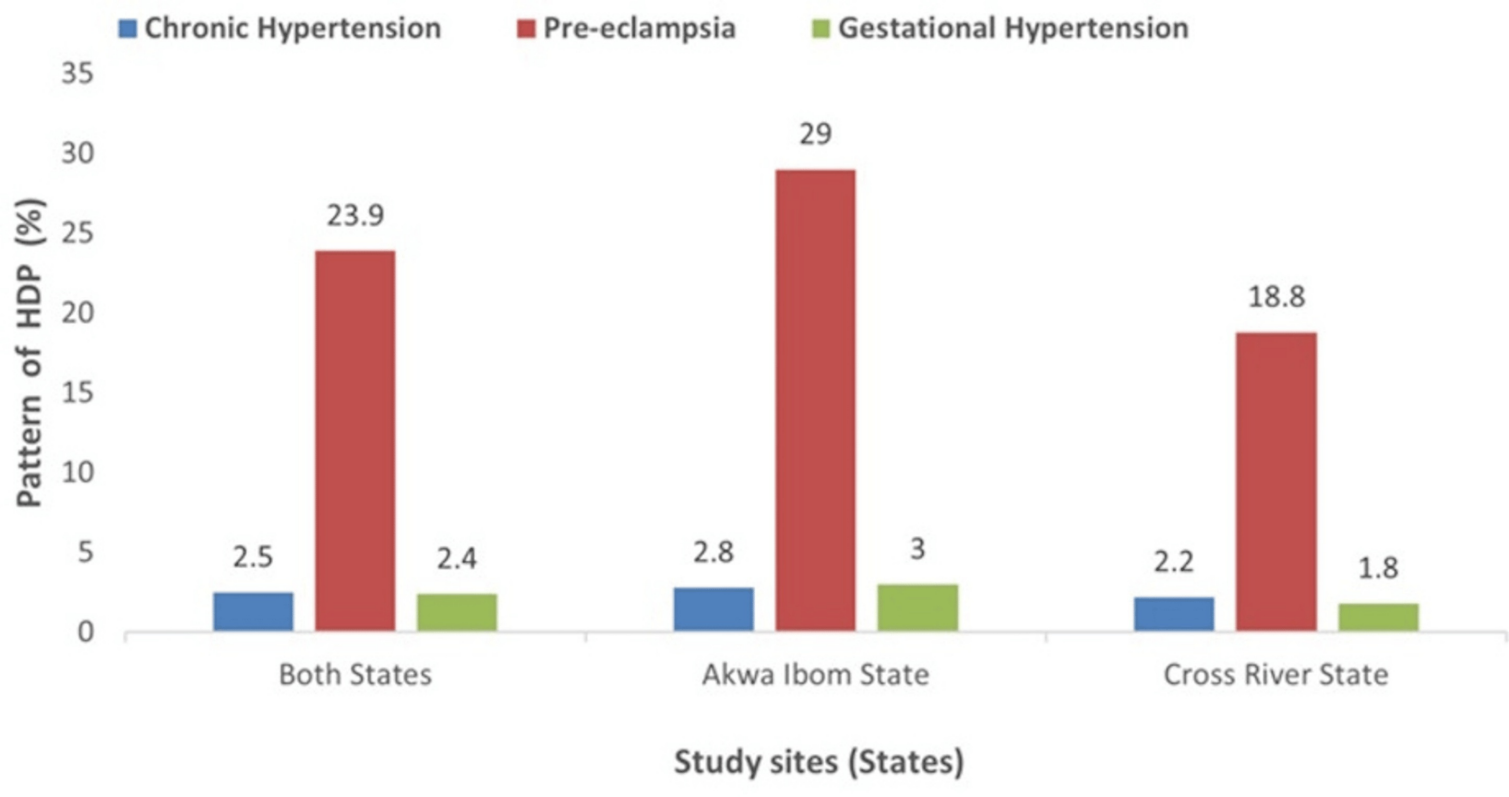
Distribution of patterns of hypertensive disorders of pregnancy among study participants. HDP: hypertensive disorders of pregnancy.

Figure 5 shows the pattern of hypertensive disorders of pregnancy in the study population and by state. Among the 1,006 participants enrolled and followed up till discharge in both Akwa Ibom and Cross River states, 25 (2.5%) were found to have chronic hypertension, while 240 (23.9%) and 24 (2.4%), respectively, were found to have pre-eclampsia and gestational hypertension. Among the 496 participants enrolled and followed up till discharge in the Akwa Ibom state study sites, 14 (2.8%) were found to have chronic hypertension, while 144 (29.0%) and 15(3.0%), respectively, were found to have pre-eclampsia and gestational hypertension. Of the 510 participants enrolled and followed up till discharge in Cross River State study sites, 11 (2.2%) were found to have chronic hypertension, while 96 (18.8%) and 9 (1.8%), respectively, were found to have pre-eclampsia and gestational hypertension.

## Discussion

Reports of incidence and prevalence of hypertensive disorders of pregnancy vary widely among different studies and across geographical regions. Higher prevalence rates have been reported in some regions of the world [11], shedding light on the urgent need for a comprehensive prospective study to unravel the complexities of this critical issue in Akwa Ibom and Cross River States. The incidence (26.9%) and prevalence (28.7%) of HDP in our study are similar to the 25.8% reported in Katsina [18] and 21.6% from South-eastern Nigeria, and also within the range of 1%-35% reported around the world [19]. However, it is higher than the 7.2% to 19.4% range found in previous studies from other Nigerian cities [18,20,21]. This high incidence of HDP in this study may not be surprising, as the present study was done in referral level hospitals, where complicated pregnancies such as HDP are referred to for management. In addition, available evidence shows that the incidence of hypertensive disorders of pregnancy has been on the increase over the years [22]. Between 1998 and 2006, in high-income countries, the prevalence of hypertension during delivery hospitalizations increased from 67.2 to 81.4 per 1000 deliveries [11]. This was ascribed in part to the increased prevalence of cardio-metabolic disease in women of childbearing age. Hence, any recent study on hypertensive disorders is likely to show increased incidence when compared to the previous studies in the same environment. The present economic downturn in Nigeria has created a lot of stress and anxiety, predisposing the citizens to hypertension, and may also explain the present high incidence of HDP [23]. In addition, the observed higher age at marriage in the part of Nigeria where this work was done, may have contributed to the increased prevalence because of the effect of age-related changes on the cardiovascular system [24].

The study also showed differences in incidence and prevalence of HDP between the two states, with Akwa Ibom State having a higher incidence and prevalence of hypertension in pregnancy than Cross River State. The varying burden of HDP in the two contiguous states is not surprising, as available evidence has shown that the burden of HDP varies not only by region but also by location, race, and ethnicity [11]. Despite the proximity of the two states, there are significant ethnic differences in the distribution of ethnic groups within them. In this study, the Ibibios constituted the largest ethnic group in Akwa Ibom State sites (56.9%), while the Efiks made up only 5%. However, ethnic diversity in Cross River State is more pronounced due to its historical significance as the former capital of both states. The Ibibios accounted for about 21.0% of the study participants in Cross River State and the Efiks made up 19.2%. Variations also exist among other ethnic groups, such as Annang, Igbo, Oro, Obolo, Ejagham, and others, in both states. In this study, it was found that 56.0% of participants with chronic hypertension at the time of booking were from Akwa Ibom State, with the remaining 44.0% from Cross River State. This suggests that hypertensive disorders are generally more prevalent in Akwa Ibom State than in Cross River State. It could be possible that the Ibibios are more prone to hypertension than the Efiks and would need to be investigated.

The pattern of HDP in this study showed pre-eclampsia (23.9%) as the most common type, followed by chronic hypertension (2.5%) and gestational hypertension (2.4%). A similar pattern was also observed in the different states. In Akwa Ibom State, pre-eclampsia (29.0%) was the most common, followed by gestational hypertension with 3.0% and chronic hypertension with 2.8%. In Cross River State, pre-eclampsia also remained the leading type of HDP with 18.8% followed by chronic hypertension, 2.2% and gestational hypertension, 1.8%. The predominance of pre-eclampsia among the types of HDP in this study was in line with 46.4% and 76.9% previously reported from Nnewi [25] and Ethiopia [26], respectively. However, studies from Bida [22] and South Africa [27] found gestational hypertension as the most common type of HDP. The finding of pre-eclampsia as the most common type of HDP in this study may have to do with the population under study. The prevalence of hypertensive disorders of pregnancy seems to be high in Akwa Ibom and Cross River states and develops early in pregnancy and those who develop gestational hypertension early in pregnancy easily convert to pre-eclampsia. Available evidence shows that when gestational hypertension occurs before 34 weeks of pregnancy, up to 35% of patients would develop pre-eclampsia [9,10]. This may explain why pre-eclampsia becomes the most common form of HDP in this environment. In addition, primigravidity (30.3%) was the preponderant gravidity group in the study population, and pre-eclampsia is known to be common among this gravidity group; hence, it may contribute to the findings in this study [28]. Pre-eclampsia, being the most common type of HDP in this environment, may have some prognostic significance as it is known that, of all the types of HDP, pre-eclampsia carries the highest maternal and fetal morbidity and mortality [9,10].

To the best of our knowledge, this is the largest prospective study, specifically on hypertensive disorders of pregnancy in Akwa Ibom and Cross River States. The prospective and meticulous nature of the data collection in this work enabled us to obtain information directly from the participants with no recall bias. However, the study had some limitations. The data were obtained from publicly funded referral-level health facilities and therefore may not reveal what is obtained in lower health facilities and the communities. In addition, there were many research assistants and in different hospitals, hence some degree of heterogeneity in the interpretation of clinical terms and accuracy in measurements (especially blood pressure) may not be completely excluded despite the initial training and the four weekly refresher virtual trainings that were mounted for them throughout the period of data collection. Furthermore, the follow-up stopped at delivery and did not extend to the postpartum period.

## Conclusions

The incidence and prevalence of HDP in Akwa Ibom and Cross River States are high and higher among the participants from Akwa Ibom State than those from Cross River State. The pattern of HDP shows that pre-eclampsia is the most common type, followed by chronic hypertension and gestational hypertension. A similar pattern is also noticed in the different states. This may have some prognostic significance, as it has been shown that of all the forms of HDP, pre-eclampsia presents with the worst prognosis. This calls for the need for our clinicians to pay close attention to the screening of our pregnant women for HDP at the antenatal clinics. Our researchers should work hard to uncover the possible predisposing factors to HDP in these states. This also throws a challenge to our obstetricians to design and implement strategies that will improve the outcomes of pre-eclampsia in these states.
